# Cultural, economic, and ecological factors influencing management of wild plants and mushrooms interchanged in Purépecha markets of Mexico

**DOI:** 10.1186/s13002-018-0269-9

**Published:** 2018-11-20

**Authors:** Berenice Farfán-Heredia, Alejandro Casas, Selene Rangel-Landa

**Affiliations:** Instituto de Investigaciones en Ecosistemas y Sustentabilidad, UNAM, Antigua Carretera a Pátzcuaro 8701, 58190 Morelia, Michoacán Mexico

**Keywords:** Biodiversity management, Biotic resources management, Cultural and economic importance, Domestication, Ecological risk, Purépecha markets

## Abstract

**Background:**

Traditional markets outstandingly contribute to conservation of biocultural diversity, social relations, and cultural values. These markets reflect life strategies and forms people of a region interact with their biodiversity and territories, as well as traditional ecological knowledge and management practices. To understand the factors motivating plant and mushroom management, we analyzed the resources cultural and economic values, their role in people’s subsistence, and the relation of these values with the resources spatial and temporal availability. Our study based on the supposition that traditional markets are settings of interchange of resources with the highest importance for people’s life in a region. Also, that the cultural, economic, and ecological factors influence values of the resources, and the demand on them determine pressures on the most valuable resources which, when scarce, motivate management innovation, otherwise become extinct.

**Methods:**

We documented cultural, economic, and ecological aspects, as well as management techniques of wild and weedy plants and mushrooms interchanged in three traditional markets of the Pátzcuaro Lake region, in central-western Mexico. For doing that, from February 2015 to March 2018, we conducted 175 visits to markets and 89 semi-structured interviews to producers, gatherers, and sellers of wild and weedy plants and mushrooms. Based on participant observation and interviews, we identified variables related to culture, economic, and ecological aspects, as well as management regimes of resources and management systems, which were documented and used as indicators for quantitative analyses. Through principal components analyses (PCA), we determined the indexes of cultural and economic importance (ICEI), management intensity (IMI), and ecological risk (IR) of the resources studied. For conducting that, we classified plant and mushroom species according to their cultural, economic, ecological, and technological indicators, respectively. The score of the first principal component was considered as the index for each group of variables, respectively. To identify relations between cultural importance and risk, we performed linear regression analyses between ICEI and IR indexes.

**Results:**

We recorded 57 species of wild and weedy plants used as food, medicine, and ornamental, and 17 species of edible mushrooms. The variables with the highest weight in the ICEI are related to the need of a resource according to people, its recognizing, the number of communities and markets offering it in markets, its explicit preference expressed by people, the effort invested in obtaining it, and the form it is interchanged. Gathering is practiced in all mushrooms and wild and weedy plants from forests and agricultural areas; 11 species in addition receive 1 or more forms of management (enhancing, selective let standing, propagation through seeds or vegetative parts, transplantation, and/or protection). The management intensity and complexity are explained by variables related to management practices and systems. Plants receiving selective management have the higher management intensity. Silvicultural management (in situ management in forests) was recorded in all species of mushrooms, as well as in more than 80% of medicinal, ceremonial and ornamental plants, and in more than 50% of the edible plants. In agricultural systems, people manage more than 90% of the edible plants recorded to be under a management regime, 25% of the managed medicinal plants, and 30.7% of the managed ceremonial and ornamental plants. In homegardens, people manage 41.6% of the medicinal plants recorded and 26.6% of the edible plants, to have them available near home. Nearly 63% of the species interchanged in the markets studied are gathered in forests without any other management form. In this group are included all mushroom species, 61.5% of ceremonial/ornamental plants, 50% of medicinal, and 33.3% of edible plants. The linear regression between ICEI an IER is significantly negative for edible species with high management intensity *R*^2^ = 0.505 (*p* = 0.0316), because of their management. But in medicinal and ornamental plants, the risk is high if the cultural importance increases, even when management practices like transplanting and propagation in homegardens are carried out.

**Conclusions:**

Traditional markets are settings of interchange of products, knowledge, and experiences, where the ongoing factors and processes motivating management innovation can be identified and documented. This approach allows documenting processes occurring at regional level but would be benefited from deeper studies at local level in communities.

## Background

Traditional markets significantly contribute to conservation of biocultural diversity, social relations, cultural values, and customs of a region. These settings reflect the life strategies, and the forms in which the cultural groups interact with their local and regional biodiversity and territories, their traditional ecological knowledge, and management practices [1–8]. In traditional markets, people interchange products using equivalence criteria according to their values, meanings, and prestige, which are necessary for reproducing life and provide cultural identity [1, 3–15]. Products interchanged in traditional markets are commonly valued based on their attributes in the cultural contexts. Such products most commonly satisfy food, medicine, ornamental purposes, and fuel, among the main needs, and the value of a species, together with its distribution and abundance, may influence the form it is managed, and the intensity, degree of specialization, and complexity of management practices [16–22]. Based on this premise, to understand the main factors that motivate plant management, it is crucial to analyze the cultural and economic values of the resources, their role in people’s subsistence, the relation of these factors with the demand of products, and the spatial and temporal availability of the resources. In addition, the relation of these factors helps to analyze their influence on the management complexity and intensity [2, 18–20, 22]. The cultural values of plant resources influence their values in the interchange, and these values in turn enhance the rate of obtaining the most valuable resources. In general, studies on these topics have found that the higher the cultural and economic value, and the lower or scarcer their availability, the higher the management intensity and complexity. When rates of obtaining resources overpass the resilience of both resources and ecosystems, such rates may determine strong risk for the maintenance of resources and/or ecosystems [16, 23]. Such conditions of risk may in turn determine uncertainty in the availability of the resources but may also enhance people to put in practice some management techniques, to ensure the resources availability [2, 3, 20–22, 24, 25]. In general, the studies analyzing these relations indicate that the higher the risk, the higher the management intensity and complexity. All these relations provide current situations about how management practices, including domestication processes, arise from situations of pressures that generate conditions of uncertainty in the access to resources. The study of these situations is thus useful for analyzing conditions and processes involved in the origins of management and domestication of biotic resources, and for reconstructing how these processes led in the past to the earliest stages of agriculture [16–27].

There are relatively few studies analyzing the influence of interchange and the increasing of extraction rates of biotic resources on the decisions to manage them, and scarce are studies directed to identify factors influencing management forms and intensities [2, 3, 20, 21, 26, 27]. But the studies available indicate that markets and patterns of interchange may determine important pressures on resources and ecosystems, and the high risk or extinction of valuable resources, which may be attenuated when people make decisions and design strategies for planning or put in practice management techniques to control such risk. Otherwise, resources extinction may take place, as it has been identified throughout the world [20–24]. Risk management is very ancient, and it is probably the primary response of humans for controlling the uncertainty of resources availability. It is probably a principal explanation of processes that led to the origins of agriculture [17–25]. Therefore, analyzing current expressions of the relation between risk and management may help to construct theory about management motives. In this case study, we aspire to contribute to this theoretical construction by documenting how people make management decisions on plants and mushrooms under pressure associated to their commercialization in markets, and its relation to their availability.

This study is based on the supposition that traditional markets are the settings of interchange of the resources with the highest importance for people’s life in a region, to which there is no direct access, or it is limited within the territory of a community. Cultural, economic, and ecological factors influence values of the resources, and the demand on them determine pressures on those resources, similarly as documented in the Tehuacán Valley and the Sierra Negra, Mexico [2, 3 18–23]. The magnitude of such pressure would be influenced by the spatial and temporal availability of a resource, and all these factors would influence the decision of people to carry out management practices. But the effectiveness of these practices also depends on the viability of managing a resource (for instance, its germination, establishment, survival, and quality in anthropogenic environments), and the history of experiences practicing management and innovation by peoples. The purposes of our study are therefore (1) to identify how all these factors interact to motivate management of wild and weedy plants and mushrooms in the Pátzcuaro Lake region, (2) to determine which species have the highest cultural and economic importance, and what factors influence their value, and (3) to know how and why the biotic resources studied are managed. Based on our previous studies, we generally hypothesized that management practices would be more intense and complex on those resources with the highest demand in markets but restricted spatial and temporal availability; contrarily, those resources abundant and with low demand in markets would receive simpler forms of management. In addition, we hypothesized that valuable scarce resources, but technically difficult to be managed would be in high risk of disappearing.

## Methods

### Study area

**Fig. 1:**
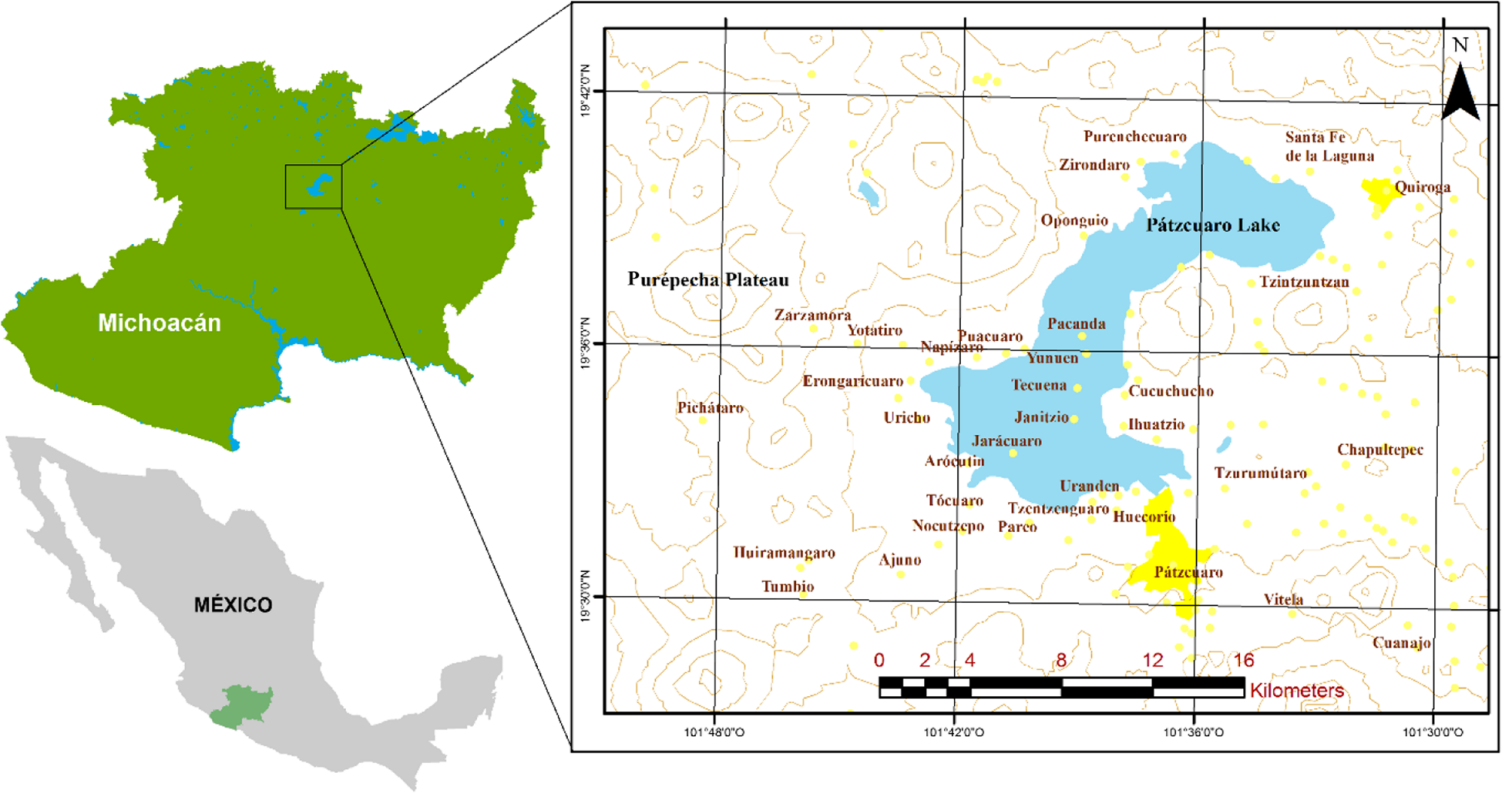
Study area. Localization of the influence region of the traditional markets, cities, and communities participating in traditional markets studied are indicated in yellow

The Pátzcuaro lake region is in the Trans-Mexican Volcanic Belt, in the state of Michoacán, Mexico (Fig. 1) at elevations from 2100 to 3280 m. Climate is temperate, sub-humid, with summer rains. Vegetation is dominated by oak, oak-pine, subtropical, and riparian forests [28].

Most communities of the region are Purépecha, which base their economy on irrigated and rainfed agriculture of maize, wheat, vegetables and fruit trees, livestock, silviculture, fishing, handcrafts making, and interchange. The region harbors high ecosystem and biocultural diversities, configuring a high heterogeneity of territories of communities that favors patterns of multiple use and management of resources and ecosystems within communities and interchange of products among them. The high heterogeneity of ecosystems in communitarian territories has historically enhanced the complementarity of resources among communities through markets [8, 29–32].

### Markets studied

**Fig. 2:**
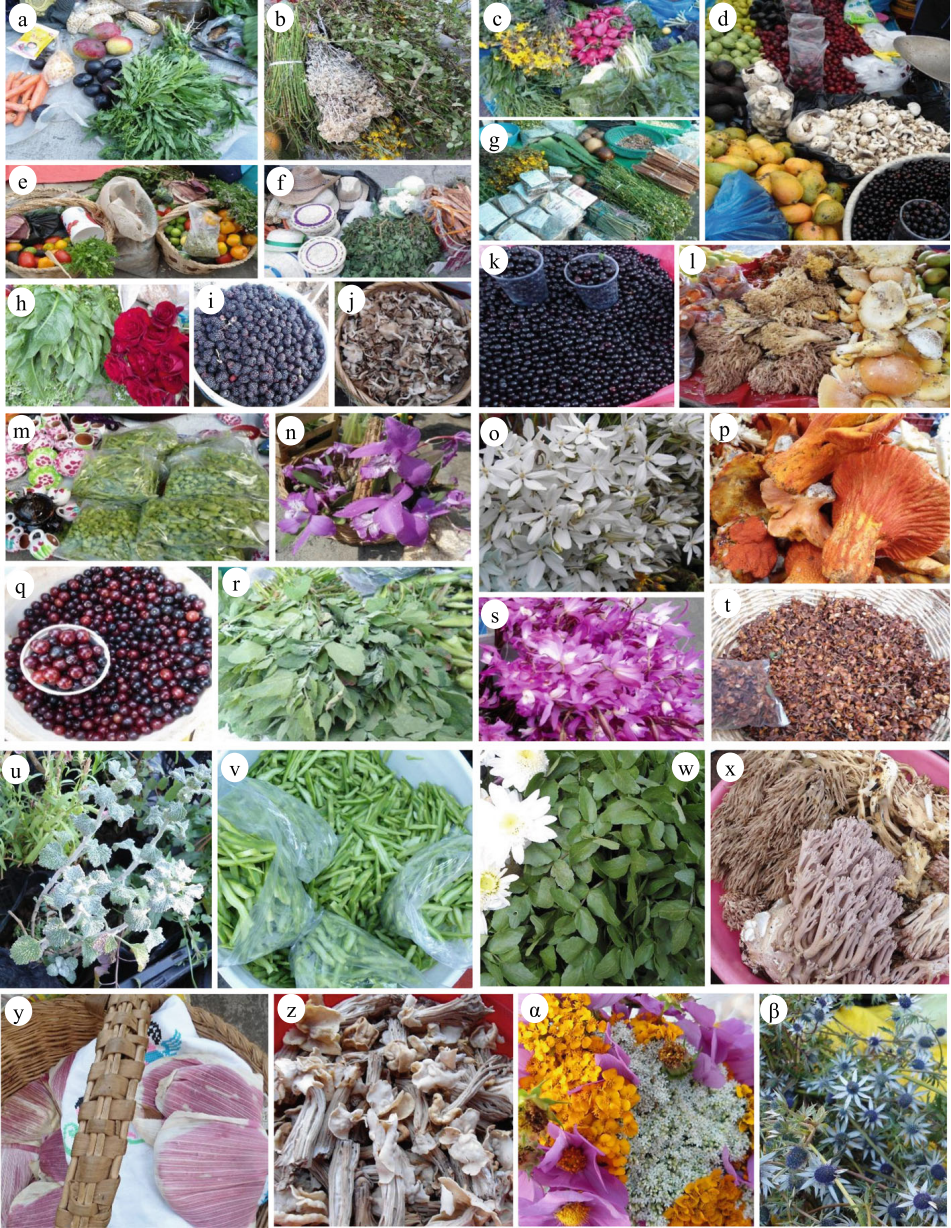
Wild and weedy plants and mushrooms interchanged in traditional markets. Diversity of edible fruits, greens, mushrooms, medicinal plants, and ceremonial and ornamental flower interchanged in the markets: **a** *Dysphania ambrosioides*; **b** medicinal plants *Equisetum* sp., *Gnaphalium* sp., *Heterotheca inuloides*, and *Clinopodium macrostemum*; **c** *Heterotheca inuloides*; **d** *Agaricus campestris* and fruits of *Prunus serotina*; **e** *Opuntia atropes* and tamal of *Rubus Liebmannii*; **f** *Chenopodium berlandieri*; **g** medicinal plants; **h** *Rumex obtusifolius*; **i** fruits of *Rubus Liebmannii*; **j** *Lyophyllum connatum* and *Lyophyllum decastes*; **k** *Prunus serotina* dark purple and big fruits variety; **l** *Hypomyces lactifluorum*, *Ramaria botrytis*, *Ramaria flavigelatinosa*, and *Amanita caesarea*; **m** *Opuntia atropes*; **n** *Laelia speciosa*; **o** *Milla biflora*; **p** *Hypomyces lactifluorum*; **q** *Prunus serotina* red and big fruits variety; **r** *Chenopodium berlandieri*; **s** *Laelia autumnalis*; **t** *Ternstroemia lineata*; **u** *Marrubium vulgare*; **v** *Opuntia atropes*; **w** *Rorippa nasturtium*-*aquaticum*; **x** *Ramaria fenica*; **y** tamales of *Rubus Liebmannii*; **z** *Helvella crispa*; **α** *Cosmos bipinnatus*, *Stevia monardifolia* and *Tagetes lucida*; **β** *Eryngium carlinae*

We studied the “Mercado de Cambio” (we will refer to it as the interchange market) located in the city of Pátzcuaro, which meets people from 29 Purépecha and Mestizo communities (from a total of 42 communities whose people arrive to the market; Fig. 2) offering wild and weedy plants and mushrooms [3, 10, 33–35]. It is carried out daily but is particularly important the Tuesdays and Fridays of every week. On average, 174 ± 34 persons arrive to this market every day, 4.8% of them for commercializing wild and weedy plants and mushrooms [3]. We also studied the “Tianguis Purépecha Regional *Mojtakuntani*” (that will be referred to as Purépecha Regional Tianguis throughout the text), which is established two Sundays per month in different places of 15 communities of the Pátzcuaro Lake shore [34, 36, 37]. On average, 28 ± 6 persons arrive per time the market is carried out, 5.5% of them selling wild and weedy plants and mushrooms (Fig. 2) [3]. And finally, we also studied the “Mercado Municipal” of the city of Pátzcuaro (ahead called Pátzcuaro Municipal Market). We conducted interviews to producers, gatherers, and sellers of the products analyzed, and we also interviewed specialized sellers who buy products that are then re-sold. On average, every day, 150 ± 27 persons arrive to this market, with 5% of them selling wild and weedy plants and mushrooms (Fig. 2) [3].

### Data collection in markets

Through participant observation and semi-structured interviews, we documented cultural and economic importance and ecological local knowledge that influence management techniques, as well as the techniques and practices involved in management of wild and weedy plants and mushrooms interchanged in markets of the Pátzcuaro Lake region. Through these interviews, we recorded information about the recognition of the resources by people, use forms and preferences of those resources, the categories of needs satisfied with the resources, gathering effort, variety of products obtained from each species, ways of interchange, value of interchange, time period of offering products in markets, community of provenance of the resources, number of markets in which the products are interchanged, plant parts gathered, perception about the resources abundance, management practices, management system, practice or not of artificial selection and the way it is practiced, and assigning categories and values to each variable (Table 1).

**Table 1:** Indicator variables of cultural, economic, ecological, and management variables used to estimate multivariate analyses

Matrix	Variables	Description	Criterion	Value
Cultural and economic variables	Recognition of species	Recognized (by 80 to 100% of interviewed persons), regularly recognized (by 40 to 79% of persons), and little recognized (by less than 40% of persons)	Recognized	3
			Regular recognized	2
			Little recognized	1
	Use form	Diversity of use forms, food is the highest value since it is the priority of people arriving to markets	Edible	2
			Medicinal	1
			Ceremonial and ornamental	1
	Use preference	Preference, according to flavor, usefulness, and its substitutability	Preferred	2
			Optional	1
	Degree of need referred to by people	According to a gradient of presence in food, traditional medicine, and the daily life of persons	Basic	4
			Complementary	3
			Sumptuous	2
			Optional-substitutable	2
			Opportunity	1
	Harvest effort invested	Effort invested in harvesting, high values are considered when gathering involves field trips exclusively planned to collect a resource	Journey dedicated to harvest the species	2
			Opportunist	1
	Variety of products	Processing at home considering a gradient of effort, time, and inputs invested	Propagated plant in pot	6
			Jam	5
			Tamales	4
			Cooked product	4
			Dehydrated product	3
			Peeled product, raw match and/or wash	2
			Bunches, bouquets, and sachets	1
	Interchange form	Diversity of interchange forms in the different contexts of the markets	Wholesale	4
			Retail sale	3
			Sale and barter	2
			Barter	1
	Interchange value	Price or monetary equivalence per selling unit. Categories of economic value are high when price is higher than $50.00 pesos, intermediate when price is from $20.00 to less than $50.00, and low when it is lower than $20.00	High	3
			Intermediate	2
			Low	1
	Period offered in markets	Period of a resource is offered, which depends on the seasonal availability but may influence the desire, need, demand, and requirement of the resources	1 to 4 months	3
			5 to 8 months	2
			9 to 12 months	1
	Number of communities offering products	Number of communities offering a product, an indicator reflecting the importance of a resource and its regional demand	9 to 15 communities	3
			5 to 8 communities	2
			1 to 4 communities	1
	Number of markets where products were recorded	Number of markets where a product is interchanged, which reflects its importance value for people’s life	Three markets	3
			Two markets	2
			One market	1
	Number of sellers	Average number of persons offering wild and weedy plants and mushrooms in the three markets	From 13 to 19	3
			From 6 to 12	2
			Less than 6	1
Ecological variables	Useful parts	According to the impact of gathering on survival, re-sprouting, and reproduction of managed populations. It was considered higher the ecological risk of gathering parts of long-life cycle individuals and mushrooms, lower risk the gathering of complete individuals of herbs, and the lowest risk the gathering of parts of herbaceous or shrubby plants	Reproductive parts	3
			Complete individual	2
			Vegetative and reproductive parts	1
	Perception of abundance	Abundance of plants and mushroom species perceived by persons interviewed	Scarce	3
			Regular abundance	2
			Abundant	1
Management variables	Management practices	Type of management practices used to increase the availability of plants and mushrooms	Propagation	6
			Transplanting of individuals	5
			Protection	4
			Enhancement	3
			Tolerance	2
			Simple gathering	1
	Management system	Type of systems where the species studied are managed, from higher to lower management intensity	Homegarden	3
			Agricultural of milpa, vegetables, and fruits	2
			Forest management	1
	Artificial selection	Presence of human selection on individual plants (not recorded any selection in mushrooms)	Selective propagation	3
			Selective tolerance	2
			Selective gathering	1
			Without selection	0

In the interchange market, we conducted 90 visits and 48 semi-structured interviews to people from 19 communities, 30 visits and 15 interviews to people from 7 communities in the Purépecha Regional Tianguis, and 55 visits and 26 interviews to people from 9 communities in the Municipal Market. All these visits and interviews were carried out from February 2015 to March 2018.

Botanical samples of plants and fungi were collected, and photographic records were made of fruits, fungi, cladodes, orchids, and products. These were deposited in the herbaria EBUM, and IEB-Bajío. The nomenclature of plant species reported follows the APG III classification system reviewed in the site http://www.theplantlist.org. Scientific names of mushrooms were consulted in the Index fungorum http://www.indexfungorum.org.

### Variables and data matrixes

Based on the interviews, we identified several cultural, economic, ecological, and management variables, which were used as indicators for quantitative analyses. The variables were categorized assigning values in gradients from higher to lower cultural and economic importance, higher to lower ecological risk, and higher to lower complexity of management practices, and all these values were defined for each species of plants and mushrooms studied (Table 1). Values for each category per species were averaged according to information from interviews (Table 6). The variables were organized in three matrixes, one with cultural and economic data, the second with ecological information, and the third one with information on management. We expected that these matrixes accounted for the main variables influencing cultural and economic importance, ecological risk and management complexity, respectively, and then we analyzed their relations.

### Data analyses

To determine the differential cultural and economic importance of the resources studied for different uses, we conducted principal components analyses (PCA) per use form and, based on the matrix with cultural and economic information, we classified the species with higher to lower importance in people’s life, identifying those variables with the higher relative influence. We considered the score of the first principal component as the index of cultural and economic importance (ICEI), since it represents the highest possible variation of the integration of all the variables analyzed. Species with the highest values were considered those with the highest cultural and economic importance, similarly as analyzed by other authors [20, 22, 26]. The PCA plot graphically represents the grouping of species per use form as a function of their relative importance according to the two first principal components.

Similarly, with the matrix containing information on management practices and systems, we conducted a PCA to classify the species according to their management intensity and complexity. We estimated the index of management intensity (IMI) based on the score of the first principal component. With the matrix of ecological information, we calculated the index of risk (IR) through the score of their PCA considering the people’s perception about resources abundance and ethnobotanical information on the parts used. We in addition conducted a linear regression analysis to identify the relation between ICEI and IER of species with high management intensity. PCA and regression analyses were carried out through JMP 11 [38].

## Results

### Cultural and economic importance of wild and weedy plants and mushrooms

#### Edible plants

We recorded 15 species of edible wild and weedy plants, 46.6% of them well recognized and appreciated by people interviewed in the markets studied; 66.6% are considered basic and complementary for subsistence (Table 6). Variation in economic and cultural importance is mainly explained by the need of a resource according to people, its recognizing, the number of persons and communities offering it in markets, the number of markets where it is found, and its explicit preference by people (46.3% of variation in the first principal component, Table 3), and the variety of products, use form, and the period the products are offered (18.5% of variation in the second principal component, Table 3).

**Fig. 3:**
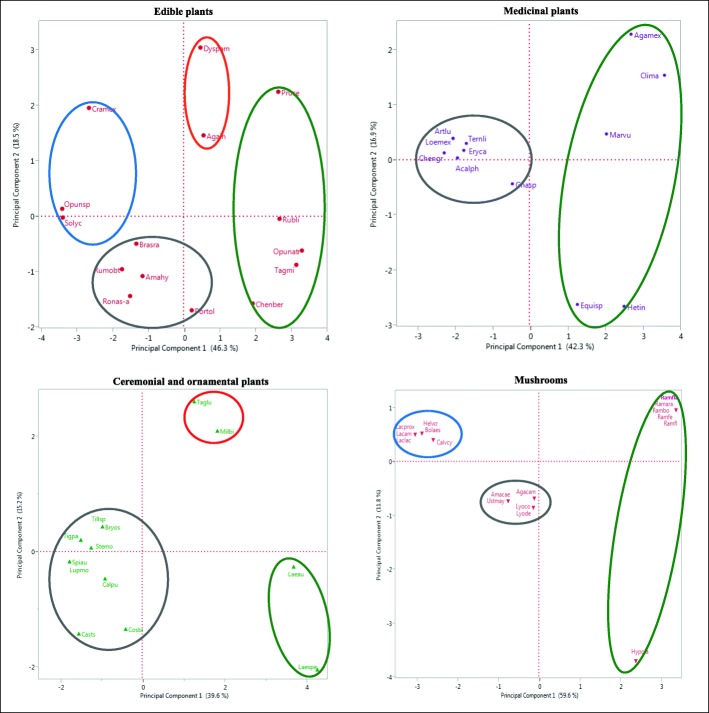
Spatial arrangement of species uses as edible, medicinal, ceremonial, and ornamental and mushrooms, according of principal component analysis performed with cultural and economic variables (for all species identity see ID correspondence on Table 2)

*Rubus liebmannii*, *Prunus serotina*, *Opuntia atropes*, *Tagetes micrantha*, and *Chenopodium berlandieri* are the edible plants with the highest cultural and economic importance, considered basic for food and subsistence, preferred over other plants, recognized by all people in markets, and offered in the three markets by the highest number of sellers from the highest number of communities (Table 2, green circles in Fig. 3). *Dysphania ambrosioides* is consumed as condiment, greens, and anthelmintic; it is considered basic and very much appreciated by people; offered for short periods in units called “manojos” (bunches), and carefully propagated in pots maintained in homegardens with special care. The scapes of *Agave inaequidens* are consumed cooked, as a sweet called “mezcal”; it is appreciated by people and offered during a short time. These two species have high scores of cultural and economic values (Table 2, red circles in Fig. 3).

**Table 2:** Cultural and economic importance (CEI), management intensity (MI), and ecological risk (ER) indexes of wild and weedy plants and mushrooms interchanged in traditional markets

Scientific name	CEI	MI	ER
Edible			
*Opuntia atropes* Rose	*3*.*319*	*0*.*116*	− 2.019
*Tagetes micrantha* Cav.	*3*.*159*	*2*.*178*	− 0.995
*Rubus Liebmannii* Focke	*2*.*699*	− 0.344	− 0.092
*Prunus serotina* subsp. *capuli* (Cav. Ex Spreng.) McVaugh	*2*.*657*	*4*.*939*	− 1.193
*Chenopodium berlandieri* Moq.	*1*.*947*	*0*.*275*	− 1.557
*Agave inaequidens* Koch	*0*.*573*	− 0.768	*1*.*010*
*Dysphania ambrosioides* (L.) Mosyakin & Clemants	*0*.*471*	*3*.*268*	*0*.*107*
*Portulaca oleracea* L.	0.241	*0*.*080*	− 1.359
*Amaranthus hybridus* L.	− 1.144	*0*.*080*	− 0.995
*Brassica rapa* L.	− 1.318	*0*.*080*	− 0.995
*Rorippa nasturtium*-*aquaticum* (L.) Hayek	− 1.493	− 0.768	− 1.898
*Rumex obtusifolius* L.	− 1.713	− 0.019	− 1.898
*Crataegus mexicana* Moc. & Sessé ex DC	− 2.634	− 0.344	*1*.*010*
*Solanum lycopersicum* L.	− 3.377	*2*.*244*	*1*.*010*
*Opuntia* sp.	− 3.388	− 0.768	*1*.*010*
Medicinal			
*Clinopodium macrostemum* (Moc. & Sessé ex Benth.) Kuntze	*3*.*603*	− 0.768	*1*.*913*
*Agastache mexicana* (Kunth) Lint & Epling	*2*.*702*	*2*.*536*	*1*.*308*
*Heterotheca inuloides* Cass.	*2*.*527*	*0*.*080*	*0*.*450*
*Marrubium vulgare* L.	*2*.*054*	*3*.*413*	*0*.*712*
*Equisetum* sp.	*1*.*274*	− 0.768	− 0.995
*Gnaphalium* sp.	− 0.455	− 0.344	− 1.546
*Ternstroemia lineata* DC	− 1.686	− 0.768	*1*.*010*
*Eryngium carlinae* F. Delaroche	− 1.748	0.080	− 0.995
*Acalypha phleoides* Cav.	− 1.922	*2*.*449*	*1*.*362*
*Loeselia mexicana* (Lam.) Brand	− 2.038	− 0.768	*0*.*812*
*Artemisia ludoviciana* Nutt.	− 2.038	− 0.768	− 1.898
*Chenopodium graveolens* Lag & Rodr.	− 2.273	*0*.*929*	*0*.*812*
Ceremonial and ornamental			
*Laelia speciosa* (Kunth) Schltr.	*4*.*273*	− 0.768	*2*.*817*
*Laelia autumnalis* (Lex.) Lindl.	*3*.*700*	− 0.768	*0*.*459*
*Milla biflora* Cav.	*1*.*837*	− 0.768	*1*.*010*
*Tagetes lucida* Cav.	*1*.*276*	*0*.*929*	− 0.092
*Cosmos bipinnatus* Cav.	− 0.398	*0*.*080*	− 1.193
*Calochortus purpureus* (Kunth) Baker	− 0.905	− 0.768	*1*.*010*
*Tillandsia* sp.	− 0.971	− 0.768	− 0.995
*Bryophyta* sensu *lato*	− 0.971	− 0.768	− 0.995
*Stevia monardifolia* Kunth	− 1.246	*0*.*929*	− 1.193
*Tigridia pavonia* (L.f.) DC.	− 1.500	*1*.*643*	− 0.995
*Castilleja scorzonerifolia* Kunth	− 1.543	− 0.768	*1*.*010*
*Lupinus montanus* Kunth	− 1.775	− 0.768	− 0.995
*Spiranthes aurantiaca (*La Llave & Lex.) Hemsl.	− 1.775	*0*.*080*	− 0.092
Mushrooms			
*Ramaria fenica* (P. Karst.) Ricken	*3*.*374*	− 0.768	− 0.092
*Ramaria flavigelatinosa* Marr & D.E. Stuntz	*3*.*374*	− 0.768	− 0.092
*Ramaria araiospora* Marr & D.E. Stuntz	*3*.*374*	− 0.768	− 0.092
*Ramaria botrytis* (Pers.) Ricken	*3*.*374*	− 0.768	− 0.092
*Ramaria flava* (Schaeff.) Quél.	*3*.*374*	− 0.768	− 0.092
*Hypomyces lactifluorum* (Schwein.) Tul. & C. Tul.	*2*.*393*	− 0.768	*1*.*010*
*Agaricus campestris* L.	− 0.100	− 0.768	− 0.092
*Lyophyllum connatum* (Schumach.) Singer	− 0.123	− 0.768	*1*.*010*
*Lyophyllum decastes* (Fr.) Singer	− 0.123	− 0.768	*1*.*010*
*Amanita caesarea* (Scop.) Pers.	− 0.751	− 0.768	*1*.*010*
*Ustilago maydis* (DC.) Corda	− 0.751	− 0.768	− 1.193
*Calvatia cyathiformis* (Bosc) Morgan	− 2.594	− 0.768	*1*.*010*
*Helvella crispa* (Scop.) Fr.	− 2.867	− 0.768	*1*.*010*
Boletus aestivalis (Paulet) Fr.	− 2.867	− 0.768	*1*.*010*
*Laccaria laccata* (Scop.) Cooke	− 3.029	− 0.768	*1*.*010*
*Laccaria amethystina* Cooke	− 3.029	− 0.768	*1*.*010*
*Laccaria squarrosa* Bandala, Montoya & Ramos	− 3.029	− 0.768	− 1.193

In italic numbers, the species with high value of cultural and economic importance, intensity of management and ecological risk

The variables with the highest weight in the ICEI are the need of a resource according to people, its recognizing, the number of persons and communities offering it in markets, the number of markets where it is found, its explicit preference by people, and the effort invested in its obtaining (Table 3).

**Table 3:** Contribution of cultural and economic variables to explain the variation of wild and weedy plants and mushrooms interchanged in traditional markets

Use type	Edible		Medicinal		Ceremonial and ornamental		Mushrooms	
Variables	PC1	PC2	PC1	PC2	PC1	PC2	PC1	PC2
Recognition	*0*.*383*	0.182	*0*.*425*	− 0.113	0.224	0.302	*0*.*322*	− 0.061
Use form	0.127	*0*.*457*	0.290	*0*.*438*	0.140	*0*.*610*	0	0
Preference	*0*.*345*	0.325	*0*.*399*	0.205	*0*.*441*	0.250	0.308	− 0.259
Need	*0*.*392*	0.006	*0*.*431*	0.105	*0*.*432*	− 0.224	0.308	− 0.259
Harvest effort	0.274	0.122	*0*.*325*	− 0.041	*0*.*455*	0.126	*0*.*329*	0.127
Variety of products	− 0.032	*0*.*486*	0.297	*0*.*337*	0	0	0	0
Form of interchange	0.214	− 0.290	0.164	− 0.517	− 0.101	*0*.*547*	*0*.*354*	0.043
Interchange value	0.184	0.123	0	0	*0*.*405*	− 0.306	0.259	− 0.079
Period offered	− 0.219	*0*.*452*	− 0.300	*0*.*429*	0	0	− 0.086	*0*.*672*
Number of communities	*0*.*305*	− 0.301	0.156	− 0.412	*0*.*407*	− 0.109	0.313	0.442
Number of markets	*0*.*359*	− 0.015	0.228	− 0.063	*0*.*407*	− 0.109	*0*.*369*	− 0.096
Number of sellers	*0*.*369*	− 0.066	0	0	0	0	0.313	0.442
Variation percentage	*46*.*3*	18.5	*42*.*2*	16.9	*39*.*6*	15.1	*59*.*6*	11.8

Data values of the first two components of the principal component analysis PC1 and PC2

Numbers in italics indicate the values of the most meaningful variables to explain the variation in each principal component

The species *Brassica rapa*, *Amaranthus hybridus*, *Rumex obtusifolius*, *Portulaca oleracea*, and *Rorippa nasturtium*-*aquaticum* are edible plants considered as optional for consumption, regularly recognized by people and with low offering (Table 2, gray circles in Fig. 3). *Crataegus mexicana*, *Opuntia* sp., and the weedy plant *Solanum lycopersicum* are consumed when there is opportunity during a short period. *C*. *mexicana* is offered prepared as jams from dehydrated and boiled fruits, offered and consumed in the communities mainly in the northern shore of the Pátzcuaro Lake (Santa Fe de la Laguna, Pátzcuaro, and San Andrés Tziróndaro (blue circles in Fig. 3).

#### Medicinal plants

We recorded 12 species of medicinal plants, 41.6% of which were recognized and said to be preferred by people in the markets; 25% were considered as basic and complementary for attending health (Table 6).

Variation in economic and cultural importance is mainly explained by the degree of the need these are considered for attending health problems, their recognizing, their preference over other species, and the effort invested in gathering them (42.2% of variation in the first principal component, Table 5) and their use form, the length of the period it is offered, and the variety of products (16.9% of variation in the second principal component, Table 3).

*Clinopodium macrostemum*, *Agastache mexicana*, *Heterotheca inuloides*, *Marrubium vulgare*, and *Equisetum* sp. are the species with the highest cultural and economic importance, considered basic and necessary; these are preferred over other medicinal plants and were recognized by all people in the markets. For obtaining them, people organize special trips to the field, except for *M*. *vulgare* which is cultivated in homegardens (Table 2, green circles in Fig. 3).

The species *Gnaphalium* sp., *Ternstroemia lineata*, *Eryngium carlinae*, *Acalypha phleoides*, *Loeselia mexicana*, *Artemisia ludoviciana*, and *Chenopodium graveolens* are medicinal plants considered optional, used when available and substitutable by other plant species, regularly recognized by people, and gathered opportunistically, when people carry out other productive activities (Table 2, gray circles in Fig. 3). Variables with the highest weight in the ICEI of medicinal plants are the degree of need these are considered for attending health problems, their recognizing, their preference over other species, and the effort invested in gathering them (Table 3).

#### Ceremonial and ornamental plants

We recorded 13 species of wild and weedy plants used for ceremonial and ornamental purposes, 38.4% of them being recognized and preferred over other species by people interviewed in the markets, 23% are considered basic and luxury, and 53.8% are considered optional or substitutable by other species. For gathering 30.7% of these plants, people organize specific journeys to the forests (Table 6). Because of their ornamental and ceremonial use, these plants have high cultural value.

Flowers of the orchids *Laelia autumnalis* and *L*. *speciosa* are recognized, considered basic, and preferred over other species for offerings during the Day of the Dead and the Corpus Christi ceremonies; for gathering these plants, people organize specific journeys; these species have the highest cultural and economic importance (Table 2, green circles in Fig. 3). *Tagetes lunata* and *Milla biflora* are ceremonial and ornamental plants, but in addition these have medicinal use (Table 2, red circles in Fig. 3).

The species *Lupinus montanus*, *Calochortus purpureus*, *Stevia monardifolia*, *Cosmos bipinnatus*, *Castilleja scorzonerifolia*, *Tillandsia* sp., *Bryophyta* sensu *lato*, *Tigridia pavonia*, and *Spiranthes aurantiaca* are plants producing showy flowers considered optional in offerings to saints, and as luxury for ornamenting houses; these are regularly recognized or identified by people, and their gathering is conducted opportunistically, when conducting other activities (Table 2, gray circles in Fig. 3).

Variables with the highest weight in the ICEI of ceremonial and ornamental plants are the gathering effort, preference over other plants, their consideration for attending a priority need, the number of communities and markets offering them (39.6% of variation in the first principal component), the forms of use and interchange (15.1% of variation in the second principal component), their interchange value, and their recognizing by people (Table 3).

#### Mushrooms

We recorded 17 species of edible mushrooms, which were recognized, preferred over other species, and considered basic for food; because of their high interchange value, the commercialization of some species contributes importantly to the economy of gatherers households; for gathering them, people organize special journeys.

*Ramaria fenica*, *R*. *flavigelatinosa*, *R*. *araiospora*, *R*. *botrytis*, *R*. *flava*, and *Hypomyces lactifluorum* are the preferred edible mushrooms, considered basic for food, with high interchange value and commercialized in high amounts to profiteers; for obtaining them, people organize special journeys to forests where the species are found. These species are offered in the three markets studied by the highest number of persons and communities. Undoubtedly, these are the species with the highest cultural and economic value (Table 2, green circles in Fig. 3).

*Lyophyllum connatum*, *L*. *decastes*, *Amanita caesarea*, *Agaricus campestris*, and *Ustilago maydis* are well recognized, high preference and considered basic by people, but are low offered in markets and their availability occurs in short periods (Table 2, gray circles in Fig. 3).

*Calvatia cyathiformis*, *Helvella crispa*, *Laccaria laccata*, *L*. *amethystina*, *Boletus aestivalis*, and *L*. *squarrosa* are little recognized mushrooms, considered optional, with low offering and interchange value, gathered only opportunistically, when people carry out other activities and find them (Table 2, blue circle in Fig. 3).

Variables with the highest weight in the ICEI of edible mushrooms are the number of markets where these are interchanged, the interchange form, gathering effort invested, recognizing of species by people (59.6% of variation in the first principal component, Table 3), the period that are offered (11.8% of variation in the first principal component, Table 3), the number of communities and people offering them, the preference over other species, and those species indicated by people as a priority need (Table 3).

#### Plant and mushrooms management

The 57 species of wild and weedy plants and mushrooms studied are gathered through different strategies, but some other management practices are additionally conducted on some species; 46 plant species (80.7%) are exclusively gathered and the other 11 species are in addition enhanced, tolerated, propagated through seeds or vegetative propagules, transplanted, and/or protected. Nearly 82.4% of the species recorded are maintained in situ in the forests, 36.8% in the multi-crop system called milpa, horticultural areas, and 15.7% in homegardens (Tables 4, 5, 7, and 8).

**Table 4:** Management practices for use form of wild and weedy plants and mushrooms interchanged in traditional markets (percentages exceed 100 because several species are under two or more management practices)

Management practice	Number of species	Percentage (%)
Gathering	57	100
Tolerance	6	10.5
Enhancement	1	1.7
Protection	1	1.7
Transplanting	5	8.7
Propagation	5	8.7

**Table 5:** Contribution of management variables to explain the variation of wild and weedy plants and mushrooms interchanged in traditional markets

Variables	PC1	PC2
Management practices	*0*.*626*	− 0.411
Management systems	*0*.*666*	− 0.158
Artificial selection	0.404	*0*.*897*
Variation percentage	*60*.*2*	*29*.*1*

Numbers in italics indicate the values of the most meaningful variables to explain the variation in each principal component

**Fig. 4:**
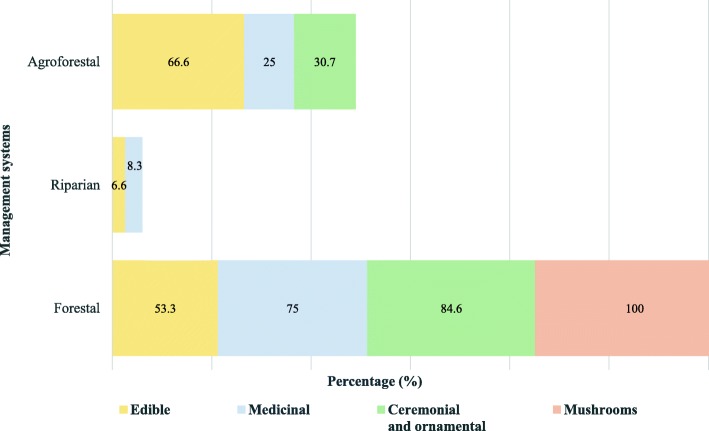
Percentage of wild and weedy plants and mushrooms by use form in management systems (percentage by use form exceed 100 because several species are distributed in two or more management system)

All the species of mushrooms analyzed are gathered in forests, without any other management practice; however, for some species, gathering is specialized and for others it is associated to opportunistic collecting, when people find them. The showy flowers of 12 species are ceremonial and ornamental, and almost all of them are exclusively gathered in forests and agricultural systems where they grow as weeds; only *Tigridia pavonia* is cultivated by seeds and by transplanting their bulbs to pots, which are commercialized in the markets when the plants bloom. Nine plant species with medicinal use, nearly 75%, are exclusively gathered in forests and agricultural systems, whereas the rest are in addition enhanced, transplanted, propagated through seeds or vegetative propagules, and/or tolerated by enhancing their availability. Nine species (60%) of the edible plants recorded are exclusively gathered, but *Prunus serotina* is managed through selective practices directed to increase the frequency of phenotypes producing dark purple and big fruits. *Rumex obtusifolius* is selectively gathered, people identify varieties called “male” and “female” varieties, the “female” variety shows desirable characteristics, and is therefore favored through different forms of management. *Tagetes micrantha* is protected and selectively gathered, favoring the small variety. The rest of the edible plant species are tolerated, enhanced, and propagated without clear signs of selection (Tables 7 and 8 and Fig. 4).

Silvicultural management (in situ management in forests) was recorded in all species of mushrooms, as well as in more than 80% of medicinal and ceremonial and ornamental plants, and more than 50% of edible plants. In the agricultural systems, people manage more than 90% of the edible plants recorded having a management form, 25% of medicinal plants, and 30.7% of ceremonial and ornamental plants. In homegardens, people manage 41.6% of medicinal plants and 26.6% of the edible plants, to have them available near home (Fig. 4).

#### Management intensity and ecological risk

**Fig. 5:**
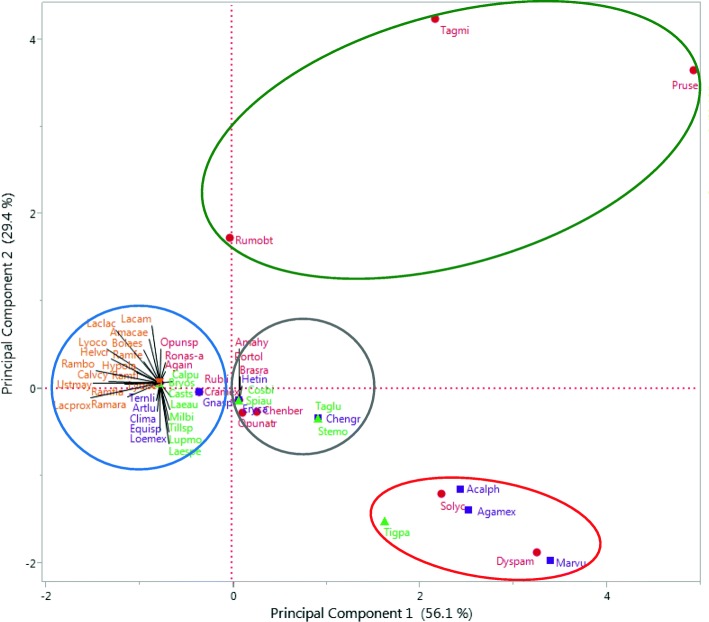
Spatial arrangement of species according of principal component analysis performed with management variables (for all species identity, see ID correspondence on Table 2, names in red are edible plants, in purple medicinal plants, in green ceremonial and ornamental plants, and in orange mushrooms)

Plants under the highest management intensity and complexity are those on which the higher number of practices, effort, and energy are invested, and are managed in different forms and in different systems, but mainly in homegardens, where the most careful forms of interaction were recorded. The management intensity and complexity are explained by the systems where plants are managed and the practices complexity (60% of variation in the first principal component), and artificial selection (29% of complexity, 60% of variation in the first principal component). In groups with higher management intensity, we identified three, two, and one species of medicinal, edible, and ceremonial/ornamental plant species, respectively (red and green circles in Fig. 5, Table 4).

Plants receiving selective management have the higher management intensity. This is the case of *Prunus serotina* in which gathering, transplanting, tolerance, and propagation in agroforestry systems is selective as explained above. Although several varieties of this species may be tolerated, people are motivated to favor the best varieties in terms of fruit quality, which have also higher demand in markets. In *Tagetes micrantha*, people distinguish the “anís chico” and “anís grande” (small and large anis, respectively) varieties, but they prefer the small variety because it is considered to have better flavor and higher density of leaves; people promote the abundance of the preferred variety in agroforestry systems. Leaves of *Rumex obtusifolius* are large, thin, and soft in the “female variety” (notice that the “female” term does not refer to sexual attributes but to a traditional classification of varieties according to their aspect, texture, and flavor), while the “male” variety has thicker, rough, and reddish leaves, and people consume only the “female” variety. The management intensity of these species is high since human selection is conducted when gathering, tolerance, enhancing, and protection are practiced (green circles in Fig. 5).

Species with low management intensity are those obtained through simple gathering. In this group, we found five edible, four ceremonial/ornamental, and three medicinal plant species (gray circles in Fig. 5).

Nearly 63% of the species interchanged in the markets studied have negative values of management intensity; these are species gathered in forests without other management. In this group are included mushrooms, 61.5% of ceremonial/ornamental plants, 50% of medicinal, and 33.3% of edible plants (blue circle in Fig. 5).

*Laelia speciosa* has the highest ecological risk value, since the complete individuals are extracted, and people perceive that it is progressively scarcer. *C*. *macrostema*, *A*. *phleoides*, and *A*. *mexicana* have also high ecological risk values because their vegetative and sexual parts, as well as their seedlings and young plants, are extracted, and people perceive that these plants are scarce and have restricted distribution (Table 2). Other plant and mushroom species that are scarce and whose individuals and reproductive structures are collected are also in risk; these are the cases of *C*. *mexicana*, *A*. *inaequidens*, *T*. *lineata*, *M*. *biflora*, and the mushrooms *L*. *connatum*, *L*. *decastes*, *A*. *caesarea*, *H*. *lactifluorum*, *L*. *laccata*, among others (Table 2). Other species whose individuals and reproductive parts are extracted but that are regularly abundant have intermediate values of ecological risk; these are the cases of *L*. *mexicana*, *C*. *graveolens*, *L*. *autumnalis*, and *H*. *inuloides*. *M*. *vulgare* is within this group since it is propagated in homegardens.

**Fig. 6:**
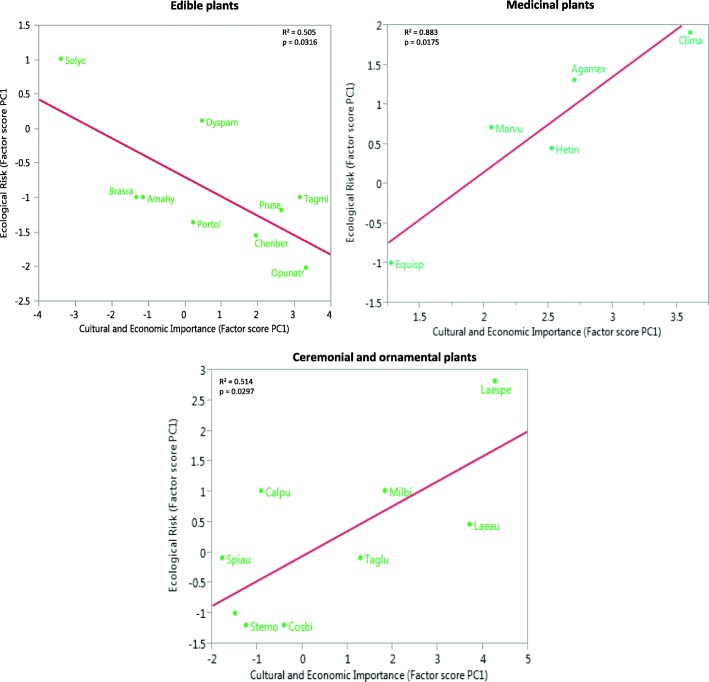
Relation between cultural and economic importance and ecological risk of species with high management intensity. Regression analysis of the ecological risk index as a function of the cultural and economic importance index

The linear regression between ICEI and IER is significantly negative for edible species with high management intensity *R*^2^ = 0.505 (*p* = 0.0316) (Fig. 6), but in the cases of medicinal and ornamental plants, the risk is high if the cultural importance increases, even when management practices like transplanting and propagation in homegardens are carried out (Fig. 6).

## Discussion

According to Casas et al. [39], decisions about how to manage plants, and most probably other biotic resources, are influenced by (1) the quality of the resources, particularly their relevance in satisfying a need and whether or not other resources may substitute a particular resource; (2) the role of resources in people’s subsistence, i.e., what needs are satisfied with them, how frequently a resource is used, and how effectively satisfy a need; (3) the amount of a resource available in the wild, a common response in the field that the authors referred to mentioned was “why to manage a resource when there is a lot available”; and (4) the viability to manage those resources, e.g., whether or not seeds germinate, how difficult is maintaining seedlings and young plants survival, how slow is plant growth, how long it takes to have available the plant products a plant species was managed for, among other common problems. All these attributes of resources were differentially represented in the groups of resources studied. Our general hypothesis relating cultural and economic importance, ecological restrictions and management, according to the results from this study should include other aspects for explaining such relation, among them (i) features related to difficulties to manage the resources, (ii) the history of management techniques versus new needs and contexts, and (iii) the differential requirements for satisfying also different needs.

The results of our study show that motivation for managing plants and mushrooms are mainly influenced by cultural and economic factors conferring value to products interchanged in markets, as well as in response to their spatial and temporal availability [3, 20–23].

### Cultural and economic importance

Variables related to the cultural and economic importance of resources are undoubtedly determinant in management decisions, to ensure availability of the most valuable resources, similarly as several authors have reported that pattern [2, 3, 20, 21, 40–42]. In the cases we studied, species with the highest cultural importance are plants and mushrooms considered indispensable, exclusive, and non-substitutable, playing crucial roles in food, traditional medicine, and rituals related to cultural identity [3, 21, 22, 40, 41]. The cultural and economic importance of a species influences its interchange value, the balance between supply and demand, and these aspects in turn influence the need of practicing management strategies to ensure benefits of interchange of money or products [2, 3, 20–22].

The scarcity or absence of a resource within a territory move people to go to markets to obtain it, in such a way that availability in markets buffers the need of managing culturally important but scarce resources. This is a strategy like others such as mobility to obtain resources within the localities or expanding their search in other regions [43], as it appears to happen in the case of mushrooms, medicinal or ceremonial plants with low management intensity. Therefore, in some contexts where a resource is scarce, the markets buffer the need of managing a culturally important but scarce resource. But in other contexts, that of the communities that satisfy the demand of a product in markets, such demand creates the need of practicing management, to increase the benefit of its commercialization or interchange. These are for instance the cases of *Renealmia alpinia and Porophyllum ruderale*, species with high commercial value in the Tehuacán Valley region, which are intensively managed ex situ to be commercialized in regional markets [20]. The species of plants and mushrooms recorded with the highest cultural value are indispensable, exclusive, and non-substitutable in food, traditional medicine, and ceremonies, but their management are also influenced by their availability and their management viability.

### Plant and mushrooms management

Gathering continues being a practice allowing access to important resources in the Pátzcuaro Lake region; all species documented in this study are gathered in forests or crop fields. Most species are obtained from forests through simple gathering, but it is possible to distinguish occasional or opportunistic gathering from planned and systematically organized gathering, mainly in mushroom gathering. In most cases, no practices directed to enhance abundance of species under organized gathering were recorded. According to people, practicing this gathering form controlling their propagation is difficult, and these are the cases of all species of mushrooms, possibly due to the difficulty of manipulating them because of their life form, their reproductive system, and the ecological interactions these organisms depend on. In addition, because the effort invested in the management could not be rewarded, since these are resources that are found in sites of common use and in many cases, there is no clear code of behavior about equitable resource acquisition [23, 25]. Perception about loss of properties and qualities of the propagated plants disincentives management. This is a phenomenon observed especially in the plants used as medicines and condiments as were the cases of *C*. *macrostemun* and *A*. *mexicana* [21]. In other cases, people say that propagation or other management forms are unnecessary since the available resources are enough for satisfying their needs. These patterns are like those documented by other authors [2, 3, 10, 20, 22, 23].

Gathering continues being the main practice for obtaining wild and weedy biotic resources in the region studied, and this practice bases on a deep traditional ecological knowledge about their biology, distribution, abundance, phenology, and morpho-physiological attributes.

### Management intensity and ecological risk

We did not identify a clear relation neither between cultural and economic importance and management intensity of the plants and mushrooms studied nor between ecological risk and management intensity. This is a pattern similarly found in the Tehuacán Valley and the Sierra Negra, Mexico with edible and medicinal plants [20, 21]. However, we did identify a significant relation between cultural and economic importance and ecological risk, particularly in plants receiving management practices like deliberate propagation, transplanting, protection, enhancing, tolerance, and gathering. In edible plants under high management intensity, we found that the higher the cultural importance, the lower the ecological risk because of the management actions. For the contrary, in medicinal, ceremonial, and ornamental plants, the higher the cultural importance, the higher the ecological risk since the latter is not counter-balanced by management practices.

Species with the highest ecological risk are those scarce, with restricted distribution, from which people extract complete individuals or reproductive structures, those having difficult propagation, or those whose qualities are lost when managed. Especially worrying are those species in which, despite the perception of their risk, people have not started regulations or management techniques for their conservation. Management techniques for those species are absent, most probably because in the past those techniques were unnecessary. Therefore, people have the challenge of starting new techniques or facing the loss of those resources.

The loss of valuable resources before over-exploitation and absence of regulations and management techniques is a frequent and extended problem among rural communities nowadays. The rhythm of innovation is therefore important. More recently, the rhythms of utilizing forest resources imposed by markets are difficult to be faced, and these are situations in which alliances between the traditional experience and scientific research are needed to find solutions. Our research group has had some experiences working with *Agave potatorum* in the Tehuacán Valley [27, 44, 45], a species occurring in dry forests. It has been over-used by people and recovery of its populations moved local people to practice seed sowing in nurseries and transplanting young plants into the field. The high mortality of plants transplanted motivated the community to ask help from our research team to solve the problem. We therefore designed studies to document the balance between availability and extraction of the resources [26], demographic studies to identify optimum harvesting, and studies on association and interactions of *A*. *potatorum* with other species in the forest [27], particularly those influencing seed germination and seedling establishment [46]. These studies in relatively short time (3 years) allowed generating new techniques that would have taken longer to be developed. We suspect that similar problems are facing those species for which management is absent however their risk to disappear. Therefore, our study allows identifying the need of studying and developing management strategies of species in critical state, such as *Laelia speciosa*, *L*. *autumnalis*, *C*. *macrostemun*, *A*. *Mexicana*, *A*. *inaequidens*, *Ramaria* spp., and *H*. *lactofluorum*.

### Our study methods

Markets are spaces where important resources are visible and are sources of important information for documenting their risk and general aspects about their management. However, these spaces alone do not allow examining in detail the factors that we hypothesize are interacting to motivate management. In those areas, we cannot see distribution and abundance of the resources studied, nor the management practices referred to by some people (who are not necessarily the resources managers) or the people interviewed underestimate some in situ management practices such as tolerance, protection, or promotion by having as reference the more complex practices such as cultivation and the former were not mentioned. Therefore, this research approach should be complemented with studies in forests, crop fields, and homegardens in the communities where people that bring resources to markets live. As demonstrated by other authors [20–22], such an approach allows evaluating amounts, frequency, and preferences of resources used by households; in addition, their distribution, abundance, and ecological contexts where the resources occur; and, finally, the details of practices carried out by people to manage those resources. What the markets allow is a regional view of what is happening with resources, and those more important at that scale. This information allows understanding the heterogeneity of the territories of the communities coexisting in a region and the complementarity needed among them. Such complementarity is increasingly important in territories that are losing common rights. Numerous communities in the Pátzcuaro Lake region and other areas of the Purépecha region of Michoacán are nowadays suffering the effect of privatizing process associated to the increasing land area for cultivating avocado, a business that is commonly associated to organized crime in Mexico. The expansion of such business is progressively canceling common rights of having access to water, forests, and even roads. If this problem increases, the traditional markets would be more important reservoirs of biocultural diversity and the opportunity for regional people to have access to traditional products. But in turn, this problem would increase pressures on wild and weedy biotic resources. Therefore, it is not only a theoretical issue involved in understanding what pressures on resources influence in terms of management motives. It is also a crucial aspect for an agenda of sustainable management of forest products in peoples’ life, in this and other regions of Mexico.

Information from the traditional markets can be complemented by studies in the communities bringing their products to those markets. Both approaches are important for understanding the reasons of socio-ecological motivations for managing biotic resources, and understanding these motivations are particularly helpful to modeling analogous factors that in the past motivated Mesoamerican people to manage and domesticate their biotic resources, thus initiating agriculture and raising of animal species.

## Conclusions

People going to traditional markets are those that strongly depend on gathered and managed resources for their subsistence, and markets are spaces where they find the products needed. Traditional markets harbor biological diversity, traditional ecological knowledge, and management techniques and are therefore important reservoirs of biocultural heritage.

Traditional markets are settings of interchange of products, knowledge, and experiences, where the processes of innovation can be documented and the factors motivating it are ongoing and can be identified.

In the region studied, all species of plants and mushrooms analyzed are mainly obtained through simple gathering, but nearly 19% of the plants recorded are also under some management practices. This is a regional indicator of what is managed and the processes that motivate management. It is probably a limited source of information compared with that which can be documented in the rural communities, but a valuable source for identifying the most meaningful resources and the relation of the demand with the most evident signs of management.

## Appendix 1

**Table 6:** Parameters used for estimating the cultural and economic importance, management intensity, and ecological risk indexes

Scientific name	ID	Use	Cultural and economic												Ecological		Management		
			Re	Uf	P	Ne	He	Vp	Fi	Iv	Po	Nc	Nm	S	Up	Ap	Mp	Ms	As
*Agave inaequidens* Koch	Again	*E*	3	2	2	3	2	6	2	1	2	1	2	1	3	3	1	1	0
*Amaranthus hybridus* L.	Amahy	*E*	2	2	1	2.5	1	1	2.5	1	2	1	2	1	2	2	1	2	0
*Brassica rapa* L.	Brasra	*E*	2	2	1	2	1	1	1	1	2	1	3	1	2	2	1	2	0
*Chenopodium berlandieri* Moq.	Chenber	*E*	3	2	1.7	3.4	1	1	2	1	1	3	3	2	1.7	1.6	1.8	2	0
*Crataegus mexicana* Moc. & Sessé ex DC	Cramex	*E*	2	2	1.5	1	1	7	1	1	3	1	1	1	3	3	1	1.5	0
*Dysphania ambrosioides* (L.) Mosyakin & Clemants	Dyspam	*E*	3	2.6	2	4	1	5.0	1	1	3	1	3	1	2	3	11.6	3	0
*Opuntia atropes* Rose	Opunatr	*E*	3	2	2	4	2.2	3	1.7	1	1	3	3	3	1	1.8	2.4	1.6	0
*Opuntia* sp.	Opunsp	*E*	1	2	1	1	1	1.6	1	1	3	1	1	1	3	3	1	1	0
*Portulaca oleracea* L.	Portol	*E*	2.3	2	1	3	1	1	2.3	1	2	3	2	2	2	1.6	1	2	0
*Prunus serotina* subsp. *capuli* (Cav. ex Spreng.) McVaugh	Pruse	*E*	3	3	2	3	1.8	1.5	2.1	2	3	2	3	3	3	1	7.3	3.4	3
*Rorippa nasturtium*-*aquaticum* (L.) Hayek	Ronas-a	*E*	1.3	2	1	2	1	1	3	1	2	1	2	1	1	2	1	1	0
*Rubus Liebmannii* Focke	Rubli	*E*	3	2	2	4	1	1	2	3	2	2	3	3	3	2	1	1.5	0
*Rumex obtusifolius* L.	Rumobt	*E*	1.4	2	1	2	1	1	1	1	2	2	2	1	1	2	1	1	1
*Solanum lycopersicum* L.	Solyc	*E*	1	2	1	1	1	1	1	1	3	1	1	1	3	3	7	3	0
*Tagetes micrantha* Cav.	Tagmi	*E*	3	2	2	3.8	2	1	2.7	1	2	3	3	3	2	2	1.4	2	2.6
*Acalypha phleoides* Cav.	Acalph	*M*	1	1	1	1	1	1	2	1	3	1	2	1	4	2.5	6	3.5	0
*Agastache mexicana* (Kunth) Lint & Epling	Agamex	*M*	3	3	2	3.3	2	2.6	1.3	1	3	1	2	1	3.3	3	8.3	3	0
*Artemisia ludoviciana* Nutt.	Artlu	*M*	1	1	1	1	1	1	1	1	3	1	2	1	1	2	1	1	0
*Chenopodium graveolens* Lag & Rodr.	Chengr	*M*	1	1	1	1	1	1	2	1	3	1	1	1	4	2	1	3	0
*Clinopodium macrostemum* (Moc. & Sessé ex Benth.) Kuntze	Clima	*M*	3	3	2	4	1.6	3	2.3	1	2	1	3	1	4	3	1	1	0
*Equisetum* sp.	Equisp	*M*	3	1	1	2	2	1	6	1	1	1	2	1	2	2	1	1	0
*Eryngium carlinae* F. Delaroche	Eryca	*M*	1	1	1	1.5	1	1	1.7	1	3	1	2	1	2	2	1	2	0
*Gnaphalium* sp.	Gnasp	*M*	2.2	1	1	1.7	1	1	1.5	1	2	1	3	1	2	1.5	1	1.5	0
*Heterotheca inuloides* Cass.	Hetin	*M*	3	1	1.8	3	1.4	1	3.4	1	1	3	3	1	3.6	2	1	2	0
*Loeselia mexicana* (Lam.) Brand	Loemex	*M*	1	1	1	1	1	1	1	1	3	1	2	1	4	2	1	1	0
*Marrubium vulgare* L.	Marvu	*M*	3	1	1.6	2.6	1	4.3	1.3	1	1	1	3	1	2.6	3	12.3	3	0
*Ternstroemia lineata* DC	Ternli	*M*	1	1	1	1	1	1	1	1	3	1	3	1	3	3	1	1	0
*Bryophyta* sensu *lato*	Bryos	*C*-*O*	3	1	1	2	1	1	3	1	3	1	1	1	2	2	1	1	0
*Calochortus purpureus* (Kunth) Baker	Calpu	*C*-*O*	1	1	1	2	1	1	3	1	3	1	2	1	3	3	1	1	0
*Castilleja scorzonerifolia* Kunth	Casts	*C*-*O*	1	1	1	1	1	1	2	1	3	1	1	1	3	3	1	1	0
*Cosmos bipinnatus* Cav.	Cosbi	*C*-*O*	2	1	1	2	1	1	2	1	3	1	2	1	3	1	1	2	0
*Laelia autumnalis (*Lex.) Lindl.	Laeau	*C*-*O*	3	1	2	6	2	1	3	2	3	1	2	1	3	2.5	1	1	0
*Laelia speciosa (*Kunth) Schltr.	Laespe	*C*-*O*	2	1	2	6	2	1	2	2	3	1	3	1	5	3	1	1	0
*Lupinus montanus* Kunth	Lupmo	*C*-*O*	1	1	1	1	1	1	3	1	3	1	1	1	2	2	1	1	0
*Milla biflora* Cav.	Mibi	*C*-*O*	3	1.5	2	2	2	1	3	1	3	1	2	1	3	3	1	1	0
*Spiranthes aurantiaca* (La Llave & Lex.) Hemsl.	Spiau	*C*-*O*	1	1	1	1	1	1	3	1	3	1	1	1	3	2	1	2	0
*Stevia monardifolia* Kunth	Stemo	*C*-*O*	2	1	1	2	1	1	3	1	3	1	1	1	3	1	1	3	0
*Tagetes lucida* Cav.	Taglu	*C*-*O*	2	2	2	2	1.5	1	3	1	3	1	2	1	3	2	1	3	0
*Tigridia pavonia (*L.f.) DC.	Tigpa	*C*-*O*	2	1	1	1	1	1	3	1	3	1	1	1	2	2	12	1	0
*Tillandsia* sp.	Tillsp	*C*-*O*	3	1	1	2	1	1	3	1	3	1	1	1	2	2	1	1	0
*Agaricus campestris* L.	Agacam	*H*	2	2	2	4	2	1	2	2	3	1	2	1	3	2	1	1	0
*Amanita caesarea* (Scop.) Pers.	Amacae	*H*	2	2	2	4	1	1	2	2	3	1	2	1	3	3	1	1	0
*Boletus aestivalis* (Paulet) Fr.	Bolaes	*H*	2	2	1	1	1	1	2	1	3	1	1	1	3	3	1	1	0
*Calvatia cyathiformis (*Bosc) Morgan	Calvcy	*H*	2	2	1	1	1	1	1	2	3	1	1	1	3	3	1	1	0
*Helvella crispa (*Scop.) Fr.	Helvcr	*H*	2	2	1	1	1	1	2	1	3	1	1	1	3	3	1	1	0
*Hypomyces lactifluorum* (Schwein.) Tul. & C. Tul.	Hypola	*H*	3	2	2	4	2	1	6	3	2	1	3	1	3	3	1	1	0
*Laccaria amethystina* Cooke	Lacam	*H*	2	2	1	1	1	1	1	1	3	1	1	1	3	3	1	1	0
*Laccaria laccata* (Scop.) Cooke	Laclac	*H*	2	2	1	1	1	1	1	1	3	1	1	1	3	3	1	1	0
*Laccaria squarrosa* Bandala, Montoya & Ramos	Lacprox	*H*	2	2	1	1	1	1	1	1	3	1	1	1	3	3	1	1	0
*Lyophyllum connatum* (Schumach.) Singer	Lyoco	*H*	3	2	2	4	1	1	2	2	3	1	2	1	3	3	1	1	0
*Lyophyllum decastes* (Fr.) Singer	Lyode	*H*	3	2	2	4	1	1	2	2	3	1	2	1	3	3	1	1	0
*Ramaria araiospora* Marr & D.E. Stuntz	Ramara	*H*	3	2	2	4	2	1	6	3	3	2	3	2	3	2	1	1	0
*Ramaria botrytis* (Pers.) Ricken	Rambo	*H*	3	2	2	4	2	1	6	3	3	2	3	2	3	2	1	1	0
*Ramaria fenica* (P. Karst.) Ricken	Ramfe	*H*	3	2	2	4	2	1	6	3	3	2	3	2	3	2	1	1	0
*Ramaria flava* (Schaeff.) Quél.	Ramfla	*H*	3	2	2	4	2	1	6	3	3	2	3	2	3	2	1	1	0
*Ramaria flavigelatinosa* Marr & D.E. Stuntz	Ramfl	*H*	3	2	2	4	2	1	6	3	3	2	3	2	3	2	1	1	0
*Ustilago maydis* (DC.) Corda	Ustmay	*H*	3	2	2	4	1	1	2	2	3	1	2	1	3	1	1	1	0

*E* edible, *M* medicinal, *C*-*O* ceremonial and ornamental, *H* mushrooms. *Re* recognition, *Uf* use form, *P* preference, *Ne* need, *He* harvest effort, *Vp* variety of products, *Fi* form of interchange, *Iv* interchange value, *Po* period offered, *Nc* number of communities, *Nm* number of markets, *S* number of sellers, *Up* useful parts, *Ap* abundance perception, *Mp* management practices, *Ms* management systems, *As* artificial selection

## Appendix 2

**Table 7:** Management of wild and weedy plants interchanged in traditional markets

Scientific names	Use form	Useful parts	Management systems	Management practices	Abundance perception	Voucher number
*Agave inaequidens* Koch	E	Escape	Forest	Gathering	Scarce	PhR
*Amaranthus hybridus* L.	E	CI	Milpa	Gathering	Regular abundance	BFH-362
*Brassica rapa* L.	E	CI	Milpa	Gathering	Regular abundance	BFH-360
*Chenopodium berlandieri* Moq.	E	CI	Horticultural areas	Gathering, tolerance, enhancing	Regular abundance	BFH-351
*Crataegus mexicana* Moc.& Sessé ex DC	E	Fruits	Milpa, fruit tres plantation, forest	Gathering	Scarce	PhR
*Dysphania ambrosioides* (L.) Mosyakin & Clemants	E	*RV*	Homegarden	Gathering, tolerance, propagation, sowing	Scarce	BFH-361
*Opuntia atropes* Rose	E	Stems	Homegarden, milpa, fruit tres plantation, Forest	Gathering, tolerance, propagation	Regular abundance	PhR
*Opuntia* sp.	E	Fruits	Forest	Gathering	Scarce	PhR
*Portulaca oleracea* L.	E	CI	Horticultural areas	Gathering	Regular abundance	BFH-365
*Prunus serotina* subsp. *capuli* (Cav. ex Spreng.) McVaugh	E, M	*RV*	Homegarden, milpa, fruit tres plantation, forest	Selective gathering, selective tolerance, selective transplanting, selective sowing	Abundant	BFH-380
*Rorippa nasturtium*-*aquaticum* (L.) Hayek	E	CI	Forest	Gathering	Regular abundance	BFH-355
*Rubus Liebmannii* Focke	E	Fruits	Forest, milpa	Gathering	Regular abundance	PhR
*Rumex obtusifolius* L.	E	Leaves	Milpa, forest	Selective gathering	Regular abundance	BFH-359
*Solanum lycopersicum* L.	E	Fruits	Homegarden	Gathering, sowing	Scarce	PhR
*Tagetes micrantha* Cav.	E	*RV*	Milpa, forest	Selective gathering, selective tolerance, protection	Regular abundance	BFH-366
*Acalypha phleoides* Cav.	M	*RV*	Homegarden, forest	Gathering, transplanting	Scarce	BFH-363
*Agastache mexicana* (Kunth) Lint & Epling	M	*RV*	Forest, homegarden	Gathering, transplanting, propagation	Scarce	BFH-353
*Artemisia ludoviciana* Nutt.	M	*RV*	Forest	Gathering	Regular abundance	BFH-374
*Chenopodium graveolens* Lag & Rodr.	M	*RV*	Homegarden	Gathering	Regular abundance	BFH-369
*Clinopodium macrostemum* (Moc. & Sessé ex Benth.) Kuntze	M	*RV*	Forest	Gathering	Scarce	BFH-354
*Equisetum* sp.	M	*RV*	Forest	Gathering	Regular abundance	BFH-357
*Eryngium carlinae* F. Delaroche	M	*RV*	Milpa, fruit tres plantation, forest	Gathering	Regular abundance	BFH-368
*Gnaphalium* sp.	M	*RV*	Milpa, forest	Gathering	Abundant	BFH-358
*Heterotheca inuloides* Cass.	M	*RV*	Forest, fruit tres plantation, homegarden	Gathering	Regular abundance	BFH-356
*Loeselia mexicana* (Lam.) Brand	M	*RV*	Forest	Gathering	Regular abundance	BFH-364
*Marrubium vulgare* L.	M	*RV*	Homegarden	Gathering, tolerance, transplanting, propagation	Scarce	BFH-352
*Ternstroemia lineata* DC	M	R	Forest	Gathering	Scarce	PhR
*Bryophyta* sensu *lato*	C-O	*RV*	Forest	Gathering	Regular abundance	PhR
*Calochortus purpureus* (Kunth) Baker	C-O	Flowers	Forest	Gathering	Scarce	BFH-371
*Castilleja scorzonerifolia* Kunth	C-O	Flowers	Forest	Gathering	Scarce	BFH-373
*Cosmos bipinnatus* Cav.	C-O	Flowers	Milpa	Gathering	Abundant	BFH-372
*Laelia autumnalis* (Lex.) Lindl.	C-O	Flowers	Forest	Gathering	Scarce	BFH-377
*Laelia speciosa* (Kunth)Schltr.	C-O	*RV*	Forest	Gathering	Scarce	PhR
*Lupinus montanus* Kunth	C-O	Flowers	Forest	Gathering	Regular abundance	PhR
*Milla biflora* Cav.	C-O, M	Flowers	Forest	Gathering	Scarce	BFH-370
*Spiranthes aurantiaca* (La Llave & Lex.) Hemsl.	C-O	Flowers	Milpa, forest	Gathering	Regular abundance	BFH-375
*Stevia monardifolia* Kunth	C-O	Flowers	Forest	Gathering	Scarce	PhR
*Tagetes lucida* Cav.	C-O, M	Flowers	Frutal, milpa, forest	Gathering	Regular abundance	BFH-367
*Tigridia pavonia* (L.f.) DC.	C-O	CI	Forest	Gathering, propagation, transplanting	Regular abundance	PhR
*Tillandsia* sp.	C-O	*RV*	Forest	Gathering	Regular abundance	PhR

*E* edible, *M* medicinal, *C*-*O* ceremonial and ornamental, *R* reproductive parts, *CI* complete indiviuals, *RV* reproductive and vegetative parts

**Table 8:** Management of wild mushrooms interchanged in traditional markets

Scientific name	Use form	Useful parts	Management system	Management practices	Abundance perception	Voucher number
*Ramaria fenica* (P. Karst.) Ricken	E	R	Forest	Gathering	Regular abundance	PhR
*Ramaria flavigelatinosa* Marr & D.E. Stuntz	E	R	Forest	Gathering	Regular abundance	BFH-H001
*Ramaria araiospora* Marr & D.E. Stuntz	E	R	Forest	Gathering	Regular abundance	PhR
*Ramaria botrytis* (Pers.) Ricken	E	R	Forest	Gathering	Regular abundance	BFH-H002
*Ramaria flava* (Schaeff.) Quél.	E	R	Forest	Gathering	Regular abundance	BFH-H003
*Lyophyllum connatum* (Schumach.) Singer	E	R	Forest	Gathering	Scarce	PhR
*Lyophyllum decastes* (Fr.) Singer	E	R	Forest	Gathering	Scarce	PhR
*Agaricuas campestris* L.	E	R	Forest	Gathering	Regular abundance	BFH-H004
*Amanita caesarea* (Scop.) Pers.	E	R	Forest	Gathering	Scarce	PhR
*Hypomyces lactifluorum* (Schwein.) Tul. & C. Tul.	E	R	Forest	Gathering	Scarce	BFH-H005
*Calvatia cyathiformis* (Bosc) Morgan	E	R	Forest	Gathering	Scarce	PhR
*Helvella crispa* (Scop.) Fr.	E	R	Forest	Gathering	Scarce	BFH-H006
*Laccaria laccata* (Scop.) Cooke	E	R	Forest	Gathering	Scarce	BFH-H007
*Laccaria amethystina* Cooke	E	R	Forest	Gathering	Scarce	PhR
*Ustilago maydis* (DC.) Corda	E	R	Forest	Gathering	Abundant	PhR
*Boletus aestivalis* (Paulet) Fr.	E	R	Forest	Gathering	Scarce	BFH-H008
*Laccaria squarrosa* Bandala, Montoya & Ramos	E	R	Forest	Gathering	Scarce	BFH-H009

*E* edible, *R* reproductive parts

## Abbreviation

UNAM: Universidad Nacional Autónoma de México
